# Independent endothelial functions of PIEZO1 and TRPV4 in hepatic portal vein and predominance of PIEZO1 in mechanical and osmotic stress

**DOI:** 10.1111/liv.15646

**Published:** 2023-06-22

**Authors:** Naima Endesh, Eulashini Chuntharpursat‐Bon, Charlotte Revill, Nadira Y. Yuldasheva, T. Simon Futers, Gregory Parsonage, Neil Humphreys, Antony Adamson, Lara C. Morley, Richard M. Cubbon, K. Raj Prasad, Richard Foster, Laeticia Lichtenstein, David J. Beech

**Affiliations:** ^1^ School of Medicine University of Leeds Leeds UK; ^2^ School of Chemistry University of Leeds Leeds UK; ^3^ Faculty of Biology, Medicine and Health University of Manchester Manchester UK; ^4^ Department of Hepatobiliary and Transplant Surgery St James's University Hospital Leeds UK

**Keywords:** arachidonic acid metabolism, calcium signalling, calcium‐permeable channel, endothelial nitric oxide  synthase, endothelium, hepatobiliary system, liver, mechanical force, nitric oxide, non‐selective cation channel, osmolality, PIEZO channel, portal vein, transient receptor potential vanilloid 4 (TRPV4) channel, vascular relaxation, vasculature contraction, vein

## Abstract

**Background & Aims:**

PIEZO1 and TRPV4 are mechanically and osmotically regulated calcium‐permeable channels. The aim of this study was to determine the relevance and relationship of these channels in the contractile tone of the hepatic portal vein, which experiences mechanical and osmotic variations as it delivers blood to the liver from the intestines, gallbladder, pancreas and spleen.

**Methods:**

Wall tension was measured in freshly dissected portal veins from adult male mice, which were genetically unmodified or modified for either a non‐disruptive tag in native PIEZO1 or endothelial‐specific PIEZO1 deletion. Pharmacological agents were used to activate or inhibit PIEZO1, TRPV4 and associated pathways, including Yoda1 and Yoda2 for PIEZO1 and GSK1016790A for TRPV4 agonism, respectively.

**Results:**

PIEZO1 activation leads to nitric oxide synthase‐ and endothelium‐dependent relaxation of the portal vein. TRPV4 activation causes contraction, which is also endothelium‐dependent but independent of nitric oxide synthase. The TRPV4‐mediated contraction is suppressed by inhibitors of phospholipase A_2_ and cyclooxygenases and mimicked by prostaglandin E_2_, suggesting mediation by arachidonic acid metabolism. TRPV4 antagonism inhibits the effect of agonising TRPV4 but not PIEZO1. Increased wall stretch and hypo‐osmolality inhibit TRPV4 responses while lacking effects on or amplifying PIEZO1 responses.

**Conclusions:**

The portal vein contains independently functioning PIEZO1 channels and TRPV4 channels in the endothelium, the pharmacological activation of which leads to opposing effects of vessel relaxation (PIEZO1) and contraction (TRPV4). In mechanical and osmotic strain, the PIEZO1 mechanism dominates. Modulators of these channels could present important new opportunities for manipulating liver perfusion and regeneration in disease and surgical procedures.

**Abbreviations**
ACh: acetylcholine
Apa: apamin
ChTx: charybdotoxin
COX: cyclooxygenase
DMSO: dimethyl sulphoxide
EDH: endothelium‐derived hyperpolarisation
IC_50_s: 50% inhibition
L‐NAME: N^ω^‐Nitro‐L‐arginine methyl ester hydrochloride
NOS: nitric oxide synthase
PE: phenylephrine
PG: prostaglandin
PLA: phospholipase A
TAM: tamoxifen
TRPV: Transient Receptor Potential Vanilloid

**Key points**

Proteins that control the liver's blood flow are important in liver physiology, liver disease, the treatment of liver disease and the recovery of the liver after medical and surgical interventions. Here we identify proteins that control the diameter of the portal vein, which controls the majority of blood flow to the liver. We show how the proteins serve different functions and that one becomes more important as mechanical and osmotic strains occur on the vessel. We show the effectiveness of activating the proteins with specific chemicals and suggest the potential for new therapeutic drugs based on these molecules to achieve new ways for beneficial liver modulation.

- PIEZO1 channel agonism causes nitric oxide synthase‐ and endothelium‐dependent relaxation of the portal vein.
- TRPV4 channel agonism causes phospholipase A_2_‐, cyclooxygenases 1 and 2‐ and endothelium‐dependent contraction of portal vein that is mimicked by prostaglandin E_2_.
- TRPV4 antagonism inhibits the effect of TRPV4 agonism but not PIEZO1 agonism, suggesting separation of TRPV4 from PIEZO1.
- Increased wall stretch and hypo‐osmolality inhibit TRPV4 while lacking effects on or amplifying PIEZO1.
- New mechanistic insights and ways to pharmacologically modulate the liver's blood flow are suggested.

## 1 INTRODUCTION

The hepatic portal vein links capillary beds, carrying blood from the microvasculatures of the gastrointestinal tract, spleen, pancreas and gallbladder to the microvasculature of the liver.^1^ It accounts for about 75% of liver blood flow, branching and feeding into the sinusoids for first‐pass metabolism, detoxification of blood contents by hepatic mechanisms and the delivery of key regulators such as glucagon to control gluconeogenesis.^2^ It is an active vessel, helping to propel or restrict blood flow to the liver and modulating changes in the liver, such as its regeneration, by altering shear stress and stimulating angiogenesis.^3^ The vein is lined by endothelium, has a medial layer containing smooth muscle cells arranged circularly and longitudinally, and is innervated.^4^, ^5^, ^6^ Its contractile properties are influenced by chemical factors including noradrenaline, acetylcholine and angiotensin II^6^ and mechanical and osmotic factors arising from changes in vessel length, wall tension, blood flow and blood water content.^7^, ^8^, ^9^, ^10^ Flow through the portal vein increases or decreases physiologically, for example, after a meal or during physical exercise.^10^ Osmolality decreases in portal blood after the drinking of water.^11^ Portal vein physiology changes in pathology and in the treatment of disease.^12^ There are rare diseases of the portal vein that include congenital portal venous shunts, aneurysms and portal vein thrombosis. Common diseases of the liver that result in cirrhosis and portal hypertension include alcoholic and non‐alcoholic steatohepatitis.^1^, ^13^ Portal pressure may also increase after liver resection and other surgeries when normal blood flow occurs into a smaller liver,^14^ potentially triggering regeneration via nitric oxide signalling.^3^

The sensing of mechanical forces in biology is multifactorial and still poorly understood.^15^ While entire cellular systems involving many proteins, lipids and other factors are likely to be important,^15^ the discovery of PIEZO proteins, recognised by the 2021 Nobel Prize for Medicine or Physiology, has suggested that proteins exist that are specialised for force detection at the core of this biology.^16^, ^17^, ^18^ The PIEZO proteins form trimeric ion channels of almost a million Daltons, each with 114 (3 × 38) membrane‐spanning segments.^19^, ^20^ They locate particularly to the plasma membrane and integrate with membrane lipids, where they respond in milliseconds to mechanical stimuli, enabling transmembrane ionic currents and raising the concentration of cytoplasmic calcium ion (Ca^2+^), the pivotal second messenger. Although it may not be a primary stimulator, decreased osmolality may activate the channels or enhance their response to mechanical force.^21^, ^22^ The PIEZO1 protein is expressed in the endothelium, where it is a critical sensor of shear stress.^23^, ^24^, ^25^, ^26^, ^27^ It integrates physiological force with vascular architecture^24^; regulates blood pressure^25^, ^27^; maintains muscle capillary density^28^; enables physical exercise performance^28^; drives angiogenesis^24^, ^29^; regulates endothelial permeability^30^, ^31^ and determines the phosphorylation and stability of endothelial nitric oxide synthase (NOS3/eNOS),^24^, ^27^, ^28^ amongst other effects.^23^ PIEZO1 is in other cell types too, including vascular smooth muscle cells^32^ and nerve endings that control blood pressure.^33^ Relevance to the hepatic vasculature is emerging. PIEZO1 is functional in microvascular endothelial cells of mouse and human livers,^24^, ^25^, ^34^ where it stimulates ADAM10 protein and NOTCH1 signalling.^34^ It is a suggested mediator of portal hypertension through the expression of the neutrophil chemoattractant CXCL1.^35^ A novel PIEZO1 agonist, Yoda2, relaxes the portal vein via endothelial PIEZO1 and NOS3 activity.^36^

Another channel linked to vascular mechanical and osmotic responses is the TRPV4 channel.^37^ Like PIEZO1, TRPV4 is a Ca^2+^ permeable, non‐selective cation channel.^37^ TRPV4 channels participate in endothelial responses to shear stress^38^, ^39^ and have many vascular roles^40^ but, contrasting with PIEZO1, they are not clear mechanical sensors but instead apparently integrate the sensing of multiple chemical factors that include arachidonic acid metabolites.^41^ In cultured human umbilical vein endothelial cells, TRPV4 is downstream of PIEZO1, activating after stimulation of phospholipase A_2_ to amplify the intracellular Ca^2+^ signal originally triggered by PIEZO1.^42^

Here we sought to determine the relevance and relationship of PIEZO1 and TRPV4 in the contractile tone of the hepatic portal vein. We find both to be functionally important and acting via endothelium‐dependent mechanisms, yet they are opposing and apparently independent in their effects. Although we expected TRPV4 to be downstream of PIEZO1, amplifying its regulation of contractile tone, we find no evidence for this. Moreover, we suggest that PIEZO1 and TRPV4 are regulated differently by mechanical and osmotic stress, with PIEZO1 predominating when there is increased mechanical and osmotic stress.

## 2 MATERIALS AND METHODS

### 2.1 Animals

Ten‐ to fourteen‐week‐old C57BL/6J male mice were used for experiments. Only male mice were used in order to reduce variability that might arise due to sex differences and the reproductive cycle. Mice were housed in GM500 individually ventilated cages (Animal Care Systems) at 21°C, 50%–70% humidity, and a 12 ‐h light/dark cycle. They had ad libitum access to the RM1 diet (Special Diet Services) with bedding from Pure'o Cell (Datesand). All work with mice occurred under the authority of the University of Leeds Animal Welfare and Ethical Review Committee and UK Home Office Project Licences P606320FB and PP8169223. The number of cage companions was up to 5. Animals were visually inspected and weighed at a minimum of weekly intervals for welfare‐related assessments. Local animal welfare advice and steps were taken in the rare cases of concern for an animal or animals. The genetically modified mice did not display any obvious adverse effects. Animals weighed 25–35 g. Genotypes were determined by a service using real‐time PCR with specific probes designed for each gene (Transnetyx). C57BL/6J mice with the PIEZO1 gene flanked with LoxP sites (PIEZO1^flox^) and tamoxifen (TAM)‐induced disruption of the PIEZO1 gene in the endothelium were described previously.^43^ In brief, PIEZO1^flox/flox^ mice were crossed with mice expressing cre recombinase under the Cadherin5 promoter (Tg(Cdh5‐cre/ERT2)1Rha) and inbred to obtain PIEZO1^flox/flox^/Cdh5‐cre mice.^43^ TAM (Sigma‐Aldrich) was dissolved in corn oil (Sigma‐Aldrich) at 20 mg mL^−1^. Mice were injected intra‐peritoneally with 75 mg kg^−1^ TAM for 5 consecutive days, and studies were performed 10–14 days later. PIEZO1^flox/flox^/Cdh5‐cre mice that received TAM injections are referred to as PIEZO1^ΔEC^. PIEZO1^flox/flox^ littermates (lacking Cdh5‐cre) that received TAM injections were the controls (control genotype). Mice were aged 10 weeks before the deletion occurred. TAM injections and genotyping were performed by a researcher (T.S.F.) independently from the myographer (N.E.), such that the genotypes were blind to the myographer. The different genotypes were studied at random as they became available, depending on the genotypic spread of each litter. Before experiments, mice were culled by cervical dislocation according to Schedule 1 procedures approved by the UK Home Office.

### 2.2 HA‐PIEZO1 mice

Mice with a hemagglutinin (HA) tag in native PIEZO1 (PIEZO1^HA^ mice) were generated by introducing the HA sequence between amino acids A2439 and D2440 by CRISPR‐Cas9. A sgRNA was selected based on proximity to the target region and low off‐targeting potential (catcgagctgcaggactgca‐agg), and an ssDNA repair template with the HA tag sequence and 60 nt flanking homology arms designed to facilitate integration of the HA tag sequence after Cas9‐induced double strand break was synthesised (Integrated DNA Technologies, with PAGE purification). sgRNA sequence was synthesised as an Alt‐R crRNA (Integrated DNA Technologies) oligo and re‐suspended in sterile Opti‐MEM (Gibco) and annealed with tracrRNA (Integrated DNA Technologies) by combining crRNA (2.5 μg) with tracrRNA (5 μg) and heated to 95°C. After annealing the complex, an equimolar amount was mixed with Cas9 recombinant protein (1500 ng) (NEB), the ssDNA repair template (final concentration 10 ng·μL^−1^) in Opti‐MEM (total volume, 15 μL) and incubated (RT, 15 min). Mouse embryos were electroporated (Nepa21 electroporator, Sonidel) using the AltR crRNA:tracrRNA:Cas9 complex (200 ng·μL^−1^; 200 ng·μL^−1^; 200 ng·μL^−1^, respectively) and the ssDNA HDR template (500 ng·μL^−1^).^44^ Zygotes were cultured overnight, and the resulting 2‐cell embryos were surgically implanted into the oviduct of day .5 post‐coitum pseudopregnant mice. After birth and weaning, genomic DNA extracted using the REDExtract‐N‐Amp™ tissue PCR kit (Sigma) was used to genotype pups by PCR using primers cgactctaactatcccactcaac and atccctctgcagtactcacc, followed by Sanger sequencing of candidate pup1. Mice were bred to obtain homozygotes for HA‐tag‐PIEZO1 (PIEZO1^HA^ mice).

### 2.3 Myography

Portal veins were isolated from mice and transferred to a gassed (95% O_2_ and 5% CO_2_) Krebs solution, which comprised (in mM): NaCl 130, KCl 4.7, CaCl_2_ 1.16, KH_2_PO_4_ 1.18, MgSO_4_ (7H_2_O) 1.7, NaHCO_3_ 14.9, EDTA .026 and glucose 5.5 (pH 7.4). Vessels were carefully cleaned of fat and connective tissue under a dissecting microscope and cut into 1 mm‐long segments. These segments were mounted on two stainless steel wires in chambers of a myograph (Multi Wire Myograph System 620 M) filled with 5 mL of Krebs solution and maintained at 37°C. The resting tension of each segment was determined by the normalisation module of Danish Myo Technology Normalisation Module LabChart 8 to achieve the tension specified in the results. This was done by gradual distention of the segments by increasing the separation of the jaws of the myograph in a stepwise manner. LabChart 8 calculated the tension‐force relationship for the portal vein segments. Then, the segments were equilibrated for 60 min prior to experiments. Contractile viability was examined by applying a 60 mM K^+^ solution prepared by exchanging NaCl with an equimolar amount of KCl; this K^+^ concentration depolarises the smooth muscle cells, opening voltage‐gated Ca^2+^ channels and thereby causing contraction, the amplitude of which was taken as a reference in some cases. Endothelial integrity was examined by adding acetylcholine (ACh) at .3, 1, 3 and 10 μM once the phenylephrine (PE) contractile response had reached its plateau. Segments were only used for investigation if they constricted in response to PE and dilated in response to ACh (unless the endothelium was deliberately removed). Endothelial denudation was achieved by luminal rubbing of the vein on a stainless‐steel cannula and the passing of air bubbles in Krebs solution through the lumen before mounting segments on wires for tension recording. Removal of endothelium was considered successful if the segment relaxed less than 10% when exposed to 10 μM ACh. The osmolality of the Krebs solution (indicated above) was 282 mOsm·kg^−1^. Hypotonicity was achieved by decreasing the concentration of NaCl by 14 mM, generating a modified Krebs solution containing (in mM): NaCl 116, KCl 4.7, CaCl_2_ 1.16, KH_2_PO_4_ 1.18, MgSO_4_ (7H_2_O) 1.7, NaHCO_3_ 14.9, EDTA .026 and glucose 5.5 (pH 7.4). The osmolality of the modified Krebs solution was 255 mOsm·kg^−1^. The tonicity of solutions was measured using a freezing‐point depression osmometer (Model 332, Advanced Instruments).

### 2.4 Immunostaining

Wild‐type (PIEZO1^WT^) or PIEZO1^HA^ mice were anaesthetised under isoflurane (5% induction and 2% maintenance). Mice were perfused via the portal vein by syringe with PBS (10 mL), followed by 4% PFA (20 mL). The aorta and portal vein of these mice were dissected. Permeabilisation and blocking of tissue were carried out using staining buffer (PBS pH 6.8, .5% Triton, .01% Na deoxycholate, 1% BSA, .02% NaN_3_, .1 mM CaCl_2_, .1 mM MgCl_2_, .1 mM MnCl_2_) containing 2% goat serum (Agilent), overnight at 4°C on an orbital shaker. Primary antibodies against CD31 (BD Pharmingen™, 550 274, 1:100) and HA (Cell Signalling mAB3724, 1:100) were diluted in a 1:1 solution of PBS: staining buffer and incubated overnight at 4°C on an orbital shaker. Aorta and portal vein tissues were rinsed in PBS with .25% Triton (6×, 15 min) at room temperature. Goat secondary antibodies (Invitrogen A21246 and A‐11006, 1:200) were diluted in a 1:1 solution of PBS staining buffer and incubated overnight at 4°C on an orbital shaker in the dark. Excess antibody was removed by washing with PBS containing .25% Triton (6×, 15 min) at room temperature (RT). Tissues were washed in PBS prior to incisions to allow whole‐mounting between a slide and coverslip using ProLong™ Gold (Invitrogen). Imaging was carried out on the LSM710 (Carl Zeiss Ltd.). Images were exported to Fiji for final processing and assembly. A linear adjustment of brightness and contrast was applied to the entire image. The intensity value for each image was normalised to the background.

### 2.5 Reagents

All chemicals except Yoda1 and Yoda2 were purchased from Sigma Aldrich and stored at −20°C. PE (phenylephrine), ACh (acetylcholine) and L‐NAME (N^ω^‐Nitro‐L‐arginine methyl ester hydrochloride) were dissolved in distilled water to make 100 mM stocks. GSK1016790A (*N*‐[(1*S*)‐1‐[[4‐[(2*S*)‐2‐[[(2,4‐Dichlorophenyl)sulfonyl]amino]‐3‐hydroxy‐1‐oxopropyl]‐1‐piperazinyl]carbonyl]‐3‐methylbutyl]benzo [*b*]thiophene‐2‐carboxamide), GSK2193874 (3‐([1,4′‐Bipiperidin]‐1′‐ylmethyl)‐7‐bromo‐N‐(1‐phenylcyclopropyl)‐2‐[3‐(trifluoromethyl)phenyl]‐4‐quinolinecarboxamide), SC‐560 (5‐(4‐Chlorophenyl)‐1‐(4‐methoxyphenyl)‐3‐(trifluoromethyl)‐1H‐pyrazole), indomethacin, celecoxib (Celecoxib, 4‐[5‐(4‐Methylphenyl)‐3‐(trifluoromethyl)‐1H‐pyrazol‐1‐yl]benzenesulfonamide) and bromoenol lactone (E‐6‐(Bromoethylene)tetrahydro‐3‐(1‐naphthyl)‐2H‐pyran‐2‐one) were dissolved in dimethyl sulphoxide (DMSO) to make stock solutions of 10 mM. Prostaglandin E_2_ (PGE_2_), apamin (Apa) and charybdotoxin (ChTx) were dissolved in distilled water to make stock solutions of 10 mM. SIN‐1 (3‐Morpholinosydnonimine, HCl) was prepared as a 20 mM stock in DMSO. Yoda1 (2‐[5‐[[(2,6‐Dichlorophenyl)methyl]thio]‐1,3,4‐thiadiazol‐2‐yl]‐pyrazine) (Tocris) stock solution was 10 mM in DMSO. Yoda2 is a 4‐benzoic acid derivative of Yoda1.^36^ It was synthesised in‐house and prepared as a 10 mM stock solution in DMSO.

### 2.6 Data analysis

Myography traces show readings taken every .5 or 1 s, smoothed with the Savitzky–Golay filter set to 70 points. Contractions are expressed as a percentage of 60 mM K^+^‐induced contraction. Relaxation responses are expressed as the percentage reversal of the phenylephrine contraction. EC_50_ (the concentration producing 50% of the maximal response) estimates from appropriately saturating concentration‐response curves were fitted with a standard Hill equation. The data are expressed as the mean ± standard deviation (SD) for at least 5 independent experiments (*n*) on portal vein segments from separate mice (e.g., *n* = 5 indicates data from 5 mice). Paired or unpaired *t*‐tests were used to compare two groups, and one‐way ANOVA followed by Tukey's test for multiple groups. Probabilities (*p*) of *p < .05, **p < .01 and ***p < .001 are indicated for statistical significance. NS indicates no significant difference. All data were analysed by OriginPro 2020 (OriginLab).

## 3 RESULTS

### 3.1 PIEZO1 causes relaxation via endothelial nitric oxide synthase

**FIGURE 1:**
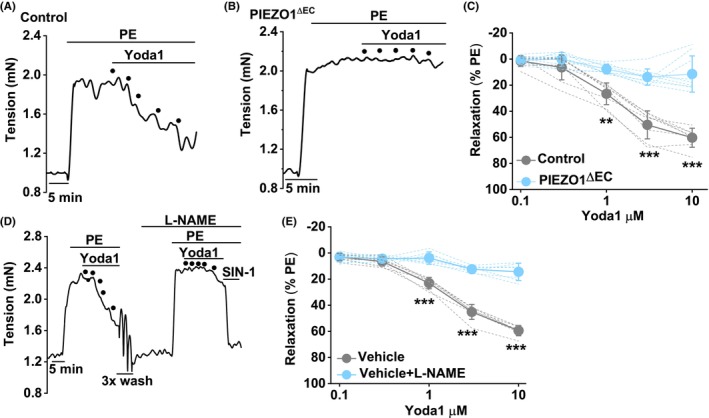
PIEZO1 agonism causes nitric oxide synthase (NOS)‐dependent relaxation. Control and gene‐modified mouse portal vein tension data (endothelium intact). (A) Example tension trace obtained from a control mouse contracted with 10 μM phenylephrine (PE) and then exposed to .1, .3, 1, 3 and 10 μM Yoda1 (PIEZO1 agonist) as indicated by the 5 dots. (B) As for (A) but from a PIEZO1^ΔEC^ mouse. (C) Summary data for Yoda1 responses of the types shown in (A, B) in *n* = 8 control mice (grey) and *n* = 9 PIEZO1^ΔEC^ mice (blue). (D) As for (A) but with a second set of concentration‐response data for Yoda1 in the presence of 100 μM L‐NAME. At the end of the recording, 10 μM SIN‐1 (a nitric oxide donor) was applied to show response to exogenous nitric oxide. Irregularities in the trace after the first Yoda1 applications occurred when the recording chamber was washed out 3 times (3× wash). (E) Summary data for *n* = 7 experiments (i.e., from 7 mice) of the type shown in (D) for the vehicle control (grey) or L‐NAME (blue). (C, E) Symbols and error bars are mean ± SD. The superimposed dotted lines are the underlying original data. Unpaired (C) and paired (D) *t*‐tests for PIEZO1^ΔEC^ compared with control mouse data at the indicated Yoda1 concentration: **p < .01, ***p < .001 and NS where there are no asterisks. *n* indicates the number of mice.

In agreement with prior findings,^36^ the PIEZO1 agonist Yoda1 causes concentration‐dependent relaxation of the portal vein pre‐contracted by α_1_‐adrenergic receptor agonist phenylephrine (PE) (Figure 1A). The effect is suppressed by conditional genetic deletion of PIEZO1 in endothelium (PIEZO1^ΔEC^) (Figure 1B,C), consistent with PIEZO1 being expressed in the endothelium of the portal vein based on immunostaining of a haemagglutinin (HA) tag incorporated in native PIEZO1^45^ (Figure S1). The generation of nitric oxide is likely to be a key event downstream of this PIEZO1 because nitric oxide synthase (NOS) inhibition by L‐NAME suppresses the effect of Yoda1 (Figure 1D,E). After endothelial deletion of PIEZO1, L‐NAME has no significant effect (Figure S2). The data suggest that activation of PIEZO1 in the endothelium causes endothelium‐ and nitric oxide‐dependent relaxation of the portal vein.

### 3.2 TRPV4 causes contraction

**FIGURE 2:**
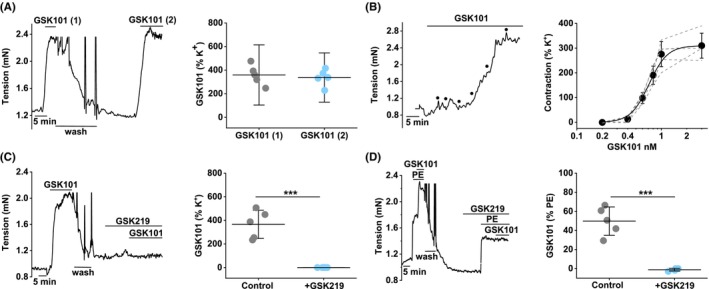
TRPV4 agonism causes contraction. Control mouse portal vein tension data (endothelium intact). (A) Example tension trace and mean summary data for contraction induced by 1 nM GSK1016790A (TRPV4 agonist) applied twice with wash‐out in between ((1) and (2)) (*n* = 5). (B) Example tension trace for contraction induced by increasing concentrations of GSK1016790A (.2, .4, .6, .8, 1 and 3 nM) as indicated by the 6 dots, with summary data to the right (*n* = 5). The smooth curve was fitted using the Hill Equation and indicated 50% maximum effect (EC_50_) at .7 nM. (C) As for (A) but with the second GSK1016790A application in the presence of 300 nM GSK2193874 (TRPV4 antagonist) (*n* = 5 for the summary data). (D) As for (C) but with GSK1016790A applied after 10 μM phenylephrine (PE). (*n* = 5 for the summary data). (A–D) Symbols and error bars are mean ± SD. The superimposed dotted lines are the underlying original data. Paired *t*‐test: (A NS), (C ***p < .001), (D ***p < .001) and NS where there are no asterisks. *n* indicates the number of mice.

Selective TRPV4 pharmacology exists.^46^, ^47^ Application of GSK1016790A, a TRPV4 agonist,^47^ in the absence of PE‐induced tone causes strong reversible contraction (Figure 2A,B), similar in magnitude and character to the contraction caused by PE (cf Figure 1A). The contraction is similar when GSK1016790A is washed out and then applied for a second time (Figure 2A). The effect is concentration‐dependent, with 50% maximum effect (EC_50_) occurring at .7 nM (Figure 2B), which is in the potency range expected for TRPV4.^47^ The GSK1016790A effect is abolished by the TRPV4 antagonist GSK2193874^46^ (Figure 2C). GSK1016790A potentiates tone in the presence of PE and this effect is blocked by GSK2193874 (Figure 2D). The data suggest that TRPV4 activation causes contraction of the portal vein.

### 3.3 TRPV4 effect depends on endothelium

**FIGURE 3:**
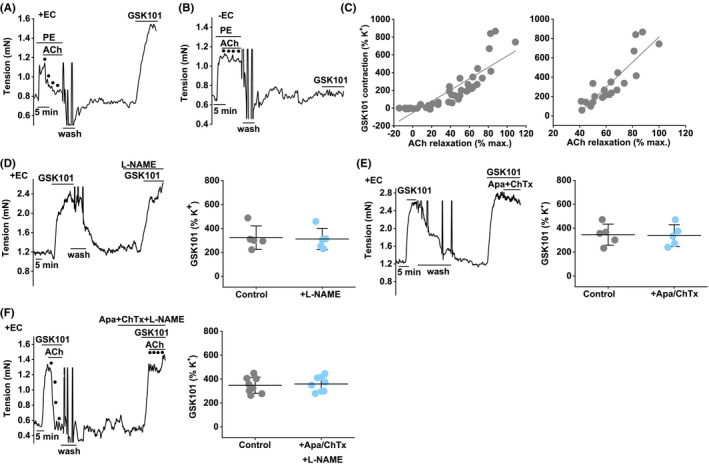
Endothelium‐dependence of the TRPV4 response. Control mouse portal vein tension data. (A) Typical trace with endothelial cells intact (+EC) showing responses to 10 μM phenylephrine (PE), .3, 1, 3 and 10 μM acetylcholine (ACh) and then, after wash out, 1 nM GSK1016790A. (B) Typical trace for a portal vein segment without endothelial cells (−EC). (C) As for (A, B), data for all experiments of this type. GSK1016790A contraction as a % of contraction evoked by 60 mM K^+^ plotted against ACh relaxation as a % of the maximum relaxation (pre‐PE tone). Each data point is for a segment of vein (*n* = 39). The straight line was fitted mathematically, indicating Pearson's correlation coefficient (*r*) .85. On the right, as for the left graph, but excluding the data in which there was no response to ACh (*n* = 21). *r* = .87. (D) For +EC, example trace (left) and summary data (right) for 1 nM GSK1016790A responses before and after incubation with 100 μM L‐NAME (*n* = 5). (E) As for (D) but using 500 nM apamin (Apa) and 100 nM charybdotoxin (ChTx) (*n* = 5). (F) As for (D, E) but using L‐NAME, Apa and ChTx (*n* = 8). Summary data are mean ± SD. Paired *t*‐test: (D–F) (NS). n indicates the number of mice.

To determine if the TRPV4 agonist (GSK1016790A) effect is endothelium‐dependent, the endothelium was physically removed. This was validated by measuring responses to acetylcholine, an endothelium‐dependent vascular relaxant.^48^ It was difficult to remove the endothelium without damaging the underlying smooth muscle, but we observed a range of acetylcholine responses and examples in which the acetylcholine response was missing yet the PE response remained (Figure 3A,B), suggesting loss of endothelial but not smooth muscle function. We plotted the amplitude of the GSK1016790A effect against the amplitude of the acetylcholine effect (Figure 3C). When there is no relaxation of acetylcholine or acetylcholine's effect is reversed into contraction, there is no effect of GSK1016790A (Figure 3C). When there is relaxation to acetylcholine, the amplitude of its relaxant response correlates positively with the amplitude of the contractile response to GSK1016790A (Figure 3C, right‐hand panel). The data suggest that the TRPV4 effect is endothelium dependent.

### 3.4 TRPV4 effect does not involve common Ca^2+^‐dependent relaxant mechanisms

TRPV4 forms Ca^2+^‐permeable channels that elevate intracellular Ca^2+^.^37^ We therefore tested for the contributions of common Ca^2+^‐dependent endothelial relaxant mechanisms, which are nitric oxide synthase 3 (NOS3) and the small and intermediate conductance Ca^2+^‐activated potassium (K) channels (SK and IK). Such mechanisms may counterbalance the contractile effect of GSK1016790A. However, the NOS3 inhibitor L‐NAME and the SK and IK inhibitors apamin and charybdotoxin have no effect on the GSK1016790A response (Figure 3D–F) despite abolishing the acetylcholine response (Figure S3). The data suggest that TRPV4 activation in the endothelium does not evoke the NOS3 or SK/IK Ca^2+^‐dependent relaxant mechanisms.

### 3.5 TRPV4 effect is inhibited by cyclooxygenase (COX) inhibitors

**FIGURE 4:**
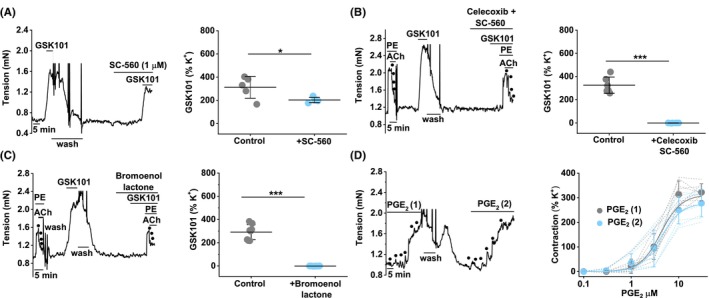
COX and PLA_2_ dependence of the TRPV4 response. Control mouse portal vein tension data (endothelium intact). (A) Example trace (left) and summary data (right) for 1 nM GSK1016790A responses before and after incubation with 1 μM SC‐560 (*n* = 5). (B) Similar to (A), but incubated with 1 μM SC‐560 and 10 μM celecoxib (*n* = 6). (C) Similar to (A), but incubated with 10 μM bromoenol lactone (*n* = 8). (D) Example trace (left) and summary data (right) for responses to increasing concentrations of prostaglandin E_2_ (PGE_2_) (.1, .3, 1, 3, 10 and 30 μM) applied twice and indicated by 6 dots each. Summary data are for n = 8 and mean ± SD. Individual data are superimposed. The two colours are for the first (1) and second (2) applications of PGE_2_ with wash out in between. Paired *t*‐test: (A *p < .05), (B, C ***p < .001) and NS where there are no asterisks. n indicates the number of mice.

COX activity is a potential mediator of portal vein contraction.^49^ COXs mediate endothelium‐derived contraction by generating prostanoids that diffuse to the smooth muscle layer.^50^ We tested if COX inhibitors affect the GSK1016790A response. SC‐560 is selective for inhibition of COX1 over COX2, with concentrations for 50% inhibition (IC_50_s) of .009 and 6.3 μM, respectively.^51^ SC‐560 (1 μM) partly inhibits the GSK1016790A response (Figure 4A) and a 10‐fold higher concentration (10 μM SC‐560) has no further effect (SI Figure S4). SC‐560 does not prevent PE‐evoked contraction or acetylcholine‐induced relaxation (Figure S4). Given that the non‐selective COX inhibitor indomethacin abolishes the GSK1016790A response (Figure S4), we hypothesised that COX2 mediates the residual response. We therefore tested a COX2‐selective inhibitor. Celecoxib (SC58635) inhibits COX1 and COX2 with IC_50_s of 15.0 and .04 μM, respectively.^52^ The combination of SC‐560 (1 μM) and celecoxib (10 μM) abolishes the GSK1016790A response (Figure 4B) without preventing PE or acetylcholine responses (Figure S4). The data suggest that COXs are required for the TRPV4‐mediated contractile effect.

### 3.6 TRPV4 effect is inhibited by phospholipase A_2_ inhibitor

The substrate for COXs is arachidonic acid generated from membrane phospholipids by Ca^2+^‐dependent phospholipase A_2_ activity.^53^ An inhibitor of phospholipase A_2_ is bromoenol lactone.^54^ Bromoenol lactone (10 μM) abolishes the GSK1016790A response (Figure 4C) without affecting PE or acetylcholine responses (Figure S4). The data suggest that phospholipase A_2_ activity mediates the TRPV4 contractile effect by generating arachidonic acid as a substrate for COX activity.

### 3.7 TRPV4 effect is mimicked by prostaglandin E_2_

COXs generate prostanoids, and several have contractile effects on smooth muscle.^50^ Prostaglandin E_2_ is found in portal vein.^55^ We tested the effect of prostaglandin E_2_ and observed concentration‐dependent contraction (Figure 4D). The EC_50_ for the prostaglandin E_2_ effect is 4 μM (Figure 4D). The data suggest that prostaglandin E_2_ is a mimic and candidate mediator of the TRPV4 contractile effect.

### 3.8 TRPV4 is not activated by PIEZO1

**FIGURE 5:**
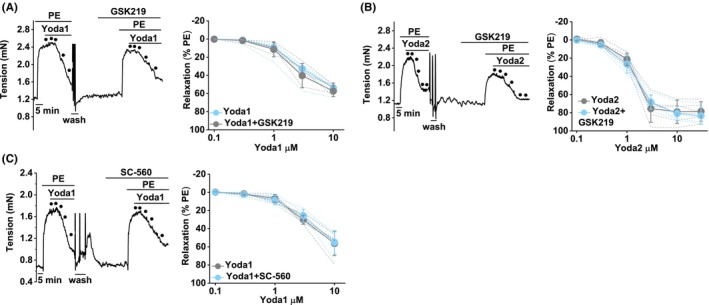
PIEZO1 responses do not involve TRPV4. Control mouse portal vein tension data (endothelium intact). (A) Example trace (left) and summary data (right) for responses to Yoda1 (.1, .3, 1, 3 and 10 μM, indicated by the 5 dots in the left trace) before and after incubation with 300 nM GSK2193874 (TRPV4 antagonist) (*n* = 5). (B) As for (A), but using Yoda2 instead of Yoda1 (.1, .3, 1, 3, 10 and 30 μM) (*n* = 5). (C) As for (A), but using 1 μM SC‐560 instead of GSK2193874 (*n* = 5). (A–C) Summary data are mean ± SD and the individual data are superimposed. Paired *t*‐tests: NS. *n* indicates the number of mice.

In cultured human umbilical vein endothelial cells, PIEZO1 activation leads to downstream TRPV4 activation.^42^ We tested if GSK2193874, the TRPV4 antagonist, affects responses to Yoda1, the PIEZO1 agonist. Yoda1‐evoked relaxation is unaffected by GSK2193874 (Figure 5A). Similarly, GSK2193874 lacks the effect on relaxation caused by Yoda2 (Figure 5B). Yoda2 is a chemical analogue of Yoda1 with improved agonist and physical and chemical properties that enable the construction of more complete concentration‐response curves and therefore EC_50_ determination.^36^ SC‐560, the COX1 inhibitor and inhibitor of the TRPV4 response (Figure 4A), also lacks an effect on the Yoda1 response (Figure 5C). The data suggest that TRPV4 and arachidonic acid metabolites do not contribute to the PIEZO1 effect on portal vein contractile tone.

### 3.9 Predominance of PIEZO1 in mechanical and osmotic strain

The portal vein experiences mechanical and osmotic variations due to events such as postprandial hyper‐perfusion of the liver, physical exercise‐dependent hypo‐perfusion of the liver and the drinking of water. To test if PIEZO1 and TRPV4 responses are affected by such events, we generated conditions in the myograph to increase vessel wall tension or lower osmolality. PE increases tension in the portal vein from about .8 to 2.2 mN (e.g., Figure 1A,D). To achieve similar tension mechanically (without applying PE), we increased basal stretch by increasing the separation between the wires in the lumen, raising basal tension from .8 ± .4 mN (Normal) to 2.2 ± .2 mN (hyper‐stretch). These tensions are approximately equivalent to those expected in response to ~6 mmHg (normal pressure)^56^ and ~15 mmHg (high pressure similar to that of portal hypertension).^57^ Acute drinking of 1 mL of water by mice lowers the osmolality of portal vein perfusate by about 25 mOsm·kg^−1^.^11^ The standard recording solution in our portal vein experiments was 282 mOsm·kg^−1^, so we lowered it to 255 mOsm·kg^−1^ (by reducing the NaCl concentration) to generate lower osmolality (Hypo‐tonicity).

**FIGURE 6:**
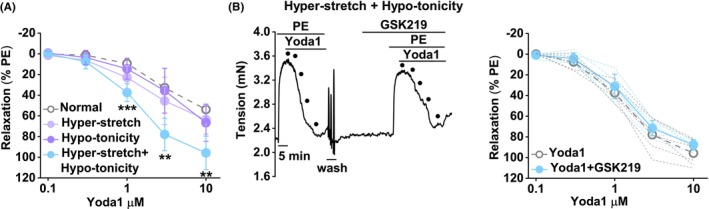
Mechanical and osmotic strain amplify PIEZO1 function. Control mouse portal vein tension data (endothelium intact). (A) Concentration‐response data for Yoda1‐induced relaxation in normal (open symbol, .8 mN tension and 282 mOsm·kg^−1^), hyper‐stretch (light purple symbol, 2.2 mN tension and 282 mOsm·kg^−1^), hypo‐tonicity (dark purple symbol, .8 mN tension and 255 mOsm·kg^−1^) and combined hyper‐stretch and hypo‐tonicity (blue symbol, 2.2 mN tension and 255 mOsm·kg^−1^) conditions (*n* = 5, 5, 5 and 6 respectively). The normal condition data are reproduced from Figure 5A and are shown only as mean values. (B) In the combined hyper‐stretch and hypo‐tonicity condition, example trace (left) and summary data (right) for responses to Yoda1 (.1, .3, 1, 3 and 10 μM, indicated by the 5 dots on the traces) before and after incubation with 300 nM GSK2193874 (TRPV4 antagonist) (*n* = 6). The Yoda1‐only data are reproduced from (a) and shown only as mean values. ANOVA (a) at the indicated Yoda1 concentration: **p < .01 and ***p < .001 for hyper‐stretch plus hypo‐tonicity compared with normal and NS, where there are no asterisks. *n* indicates the number of mice.

**FIGURE 7:**
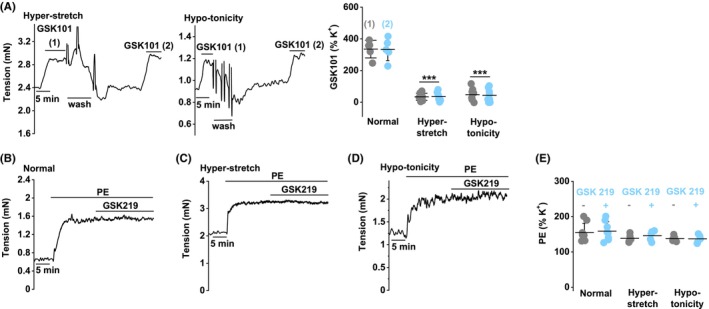
Mechanical and osmotic strain suppress TRPV4 function. Control mouse portal vein tension data (endothelium intact). (A) In the hyper‐stretch or hypo‐tonicity condition, example traces (left and middle) and summary data (right) for responses to 1 nM GSK1016790A (*n* = 8 each). The summary data are for the first (1) and second (2) GSK1016790A responses, with wash‐out in between. The normal condition data are reproduced from Figure 2A for direct comparison. (B–E) In the normal, hyper‐stretch or hypo‐tonicity condition, example traces and summary data for PE (10 μM) responses without (−) and with (+) 300 nM GSK2193874 (*n* = 8 normal, *n* = 5 hyper‐stretch, *n* = 5 hypo‐tonicity). Summary data are shown as mean ± SD. ANOVA: (A ***p < .001; hyper‐stretch or hypo‐tonicity compared with normal for (1) and (2)) and (E NS). *n* indicates the number of mice.

Hyper‐stretch alone may slightly increase Yoda1‐ and Yoda2‐evoked (PIEZO1) relaxations; hypo‐tonicity has no effect, but the combination significantly amplifies the relaxation (Figure 6A, Figure S5). As in normal conditions (Figure 5A,B), the TRPV4 antagonist (GSK2193874) does not affect Yoda1 responses in the hyper‐stretch and hypo‐tonicity conditions (Figure 6B, Figure S6). Hyper‐stretch or hypo‐tonicity strongly suppress GSK1016790A‐evoked (TRPV4) contractions (Figure 7A cf Figure 2A). A reason for such suppression could be that TRPV4 channels are already activated by the hyper‐stretch or hypo‐tonicity, but this is not the case because the TRPV4 antagonist, GSK2193874, does not affect tone in either condition (in the absence of GSK1016790A) (Figure 7B–E). Responses to 60 mM K^+^, PE or ACh are not affected by hyper‐stretch or hypo‐tonicity (Figure S7). The data suggest that hyper‐stretch and hypo‐tonicity cause a switch away from the functional relevance of TRPV4 to the predominance of PIEZO1.

## 4 DISCUSSION AND CONCLUSIONS

The results of this study suggest that PIEZO1 and TRPV4 are functionally relevant in portal vein contractile tone and have opposing independent effects of promoting relaxation and contraction, respectively, as summarised in the Figure S8. Both effects are endothelium‐dependent but via different mechanisms, which are nitric oxide synthase for PIEZO1 and arachidonic acid metabolism for TRPV4. In mechanical and osmotic stress, the PIEZO1 effect increases and the TRPV4 effect decreases, leading to the predominance of PIEZO1. The different functional effects are surprising for channels that are similarly Ca^2+^ permeable and non‐selectively permeable to cations. A potential explanation is functional separation, such as compartmentalisation, of the channels in the endothelium. The suggestion from studies of human umbilical vein endothelial cells that TRPV4 is downstream of PIEZO1 and therefore coupled^42^ is not relevant to portal vein contraction as TRPV4 inhibition does not affect PIEZO1 agonist responses. In conditions of mechanical and osmotic stress, the PIEZO1 relaxant effect is amplified and the TRPV4 contractile effect suppressed, leading to the predominance of PIEZO1 and greater potential for portal vein dilation and increased portal blood flow to the liver.

The presence of PIEZO1 in endothelium and its mediation of nitric oxide synthase activation and vessel relaxation is consistent with prior work showing that PIEZO1 agonism causes endothelial nitric oxide synthase (NOS3) phosphorylation, nitric oxide production, stabilisation of NOS3 and nitric oxide synthase‐dependent relaxation in intact artery, vein and microvasculature.^23^, ^24^, ^27^, ^28^, ^58^, ^59^ PIEZO1 channels do, however, create a dichotomy for endothelium because they are Ca^2+^‐permeable non‐selective cation channels, the activation of which causes both intracellular Ca^2+^ elevation and membrane depolarisation.^43^ Ca^2+^ elevation is associated with NOS3 activation, but depolarisation opposes endothelial hyperpolarisation, which is a relaxant mechanism often referred to as endothelium‐derived hyperpolarisation (EDH).^60^ Therefore, PIEZO1 can cause contraction by opposing EDH.^43^ The relative significance of PIEZO1's pro‐NOS3 (relaxant) and anti‐EDH (contractile) effects is likely to vary depending on the blood vessel type and context.^43^ The anti‐EDH mechanism may have little or no relevance to contractile function if gap junctions between endothelial cells and underlying smooth muscle cells are low in number, non‐functional or absent, as gap junctions transmit EDH to smooth muscle cells for functional effect. In the portal vein, gap junctions are sparse and evident only between smooth muscle cells.^4^

TRPV4 mediates endothelium‐dependent contraction of the aorta in normotensive and hypertensive animals, also via COX mechanisms, showing increased impact with ageing.^61^, ^62^, ^63^, ^64^ There is evidence of the dichotomy here too, with TRPV4‐mediated relaxant effects occurring, for example in coronary arterioles.^65^ The relationships of such effects to PIEZO1, other than what we show here for the portal vein, are unknown.

Amplification of the PIEZO1 response by hyper‐stretch and hypo‐tonicity is consistent with prior knowledge of PIEZO1 because both factors amplify PIEZO1 activity.^18^, ^21^, ^22^ Yoda1 effects synergise with effects of mechanical force.^66^ The suppression of TRPV4 responses by these factors may be due to effects indirectly associated with TRPV4 rather than effects directly on TRPV4 itself. The switch to dominance of PIEZO1 suggests that there is likely to be increased portal vein relaxation in these conditions. In this study, the portal vein was investigated in isolation; as such, the impact of blood flowing through the vessel, as well as endocrine and neurogenic effects, may alter the response to hypotonicity in vitro and in the in vivo hepatic circulation.^7^, ^8^

The TRPV4 antagonist, GSK2193874, is a good tool for exploring TRPV4 biology in vitro and in vivo.^46^ It has cross‐species potency at TRPV4, efficacy against TRPV4 channels that are activated by diverse stimuli and apparent selectivity for TRPV4.^46^ In a rat model of heart failure, GSK2193874 prevents and reverses pulmonary oedema, acting only at pathological pulmonary venous pressure.^67^ An analogue of GSK2193874 shows further benefit in pulmonary oedema in congestive heart failure.^68^ Small molecules of this type therefore have therapeutic potential. These data and our new findings suggest that it would be worth exploring TRPV4 in other conditions of high pressure, such as portal hypertension. We found TRPV4 to be less active in acute mechanical and osmotic stress, but its roles in portal pathologies are currently unknown. The strong effect of celecoxib against TRPV4‐mediated contraction in the portal vein should be considered in the context of evidence of its potential value in the treatment of portal hypertension and liver cirrhosis.^69^, ^70^, ^71^ Inhibition of TRPV4 activation could be a contributor to celecoxib's effects in these situations.

The mechanisms by which the effects of Yoda1 synergise with those of mechanical and osmotic stress are unknown. It would be helpful to know the mechanisms because we might then be better informed about the contexts in which PIEZO1 agonism is likely to be most effective, which we speculate might be in ageing and diseases such as hypertension. Results of in vitro PIEZO1 overexpression studies have suggested synergism at the level of the PIEZO1 channel itself,^66^ potentially with Yoda1 acting as a “molecular wedge” that lowers PIEZO1 sensitivity to mechanical force.^72^ PIEZO1 may, however, achieve mechanical sensitivity through multiple mechanisms that include its interaction with cytoskeletal and extracellular matrix proteins and cell–cell junction proteins such as adhesion molecules.^18^, ^45^, ^73^, ^74^ Therefore, multiple factors may explain Yoda1's increased effect in mechanical and osmotic stress. Specific investigations of this in portal pathologies such as portal hypertension would be particularly valuable. At present, we lack information on what happens to the expression or function of PIEZO1 or TRPV4 in such pathologies.

In conclusion, we reveal the opposing independent roles of PIEZO1 and TRPV4 in the regulation of portal tone and suggest the predominance of PIEZO1 in conditions of mechanical and osmotic stress. PIEZO1 is therefore likely to have particular significance in controlling first‐pass metabolism, detoxification of blood contents and gluconeogenesis. While PIEZO1 may be capable of signalling to TRPV4 in other endothelial situations, this is not relevant to portal contraction. The channels are remarkably separate despite sharing endothelial dependence. In the future, it will be important to determine the roles of PIEZO1 and PIEZO1 agonism in human hepatic vasculature, especially in conditions of increased pressure and lower osmolality, as can occur in liver disease, liver surgery and excessive drinking of water. Such studies could lead to important new opportunities for modulating liver perfusion and improving liver regeneration after disease‐related injury or surgery.

## AUTHOR CONTRIBUTIONS

Naima Endesh planned and coordinated experimental work, performed experiments, did data analysis, prepared figures and co‐wrote the paper. Nadira Y. Yuldasheva and Gregory Parsonage provided technical assistance. Eulashini Chuntharpursat‐Bon performed the microscopy experiments. T. Simon Futers and Laeticia Lichtenstein bred and maintained genetically engineered mice according to Home Office Licence requirements. T. Simon Futers coordinated genotyping and performed TAM injections. Laeticia Lichtenstein perfusion‐fixed mice. Charlotte Revill performed chemical synthesis and validation. Neil Humphreys and Antony Adamson designed and generated PIEZO1^HA^ mice. Lara C. Morley, Richard M. Cubbon and K. Raj Prasad provided intellectual input. Richard Foster led the chemical synthesis and generated funds for the chemistry. David J. Beech initiated the project, generated research funds and ideas, led and coordinated the project, interpreted data and co‐wrote the paper.

## FUNDING INFORMATION

The work was supported by research grants from Wellcome (grant number 110044/Z/15/Z) and British Heart Foundation (grant number RG/17/11/33042). For the purpose of Open Access, the authors have applied a CC BY public copyright licence to any Author Accepted Manuscript version arising from this submission.

## CONFLICT OF INTEREST STATEMENT

The authors declare no conflicts of interest other than obligations from their research grants (Financial Support).

## EXPERIMENTAL ANIMAL STUDIES

All work with mice occurred under the authority of the University of Leeds Animal Welfare and Ethical Review Committee and UK Home Office Project Licences P606320FB and PP8169223. All animals received human care, and the study protocols comply with our institution's guidelines. Animal experiments conformed to the Animal Research: Reporting of In Vivo Experiments (ARRIVE) guidelines (http://www.nc3rs.org.uk/arrive‐guidelines), developed by the National Centre for the Replacement, Refinement and Reduction of Animals in Research (NC3Rs) to improve standards and reporting of animal research.

## Supporting information


Figure S1‐S8

## Data Availability

The data that support the findings of this study are available from the corresponding author upon reasonable request.
