# Supplementary figures and images for: Independent endothelial functions of PIEZO1 and TRPV4 in hepatic portal vein and predominance of PIEZO1 in mechanical and osmotic stress
Source: Liver Int. 2023 Jun 22;43(9):2026–38. doi: 10.1111/liv.15646 (PMC10946873; doi:10.1111/liv.15646)

## SI Figure S1

**a**

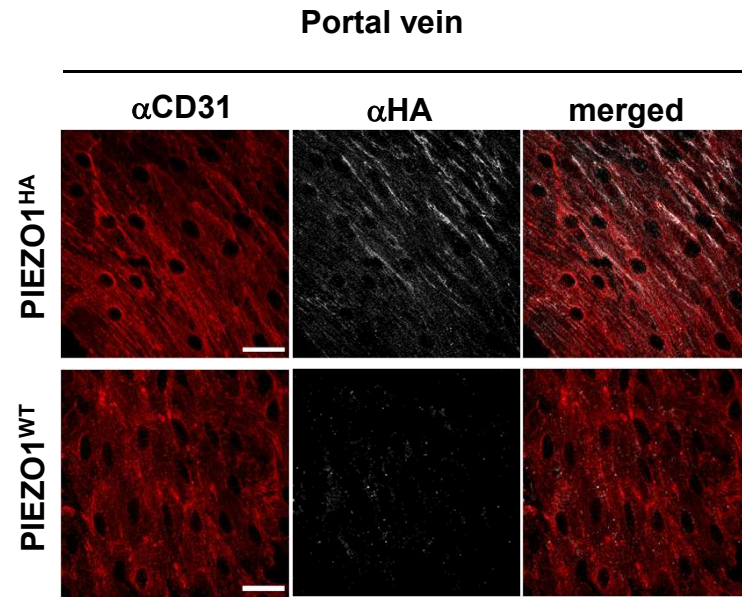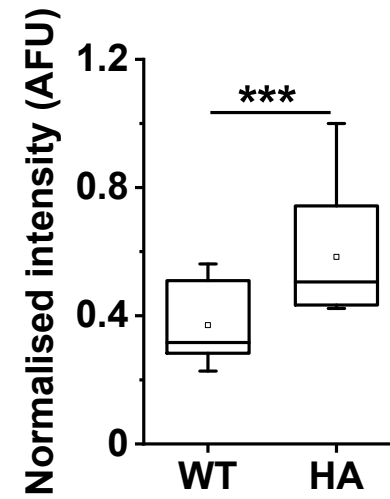

**b**

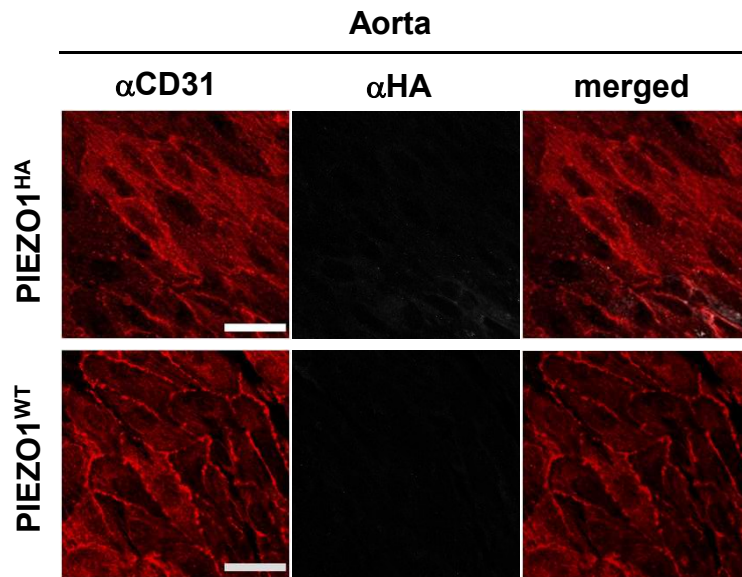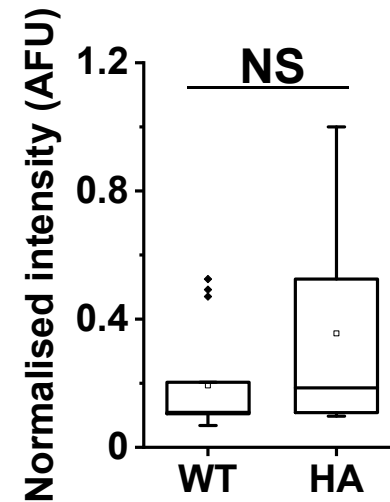

## SI Figure S2

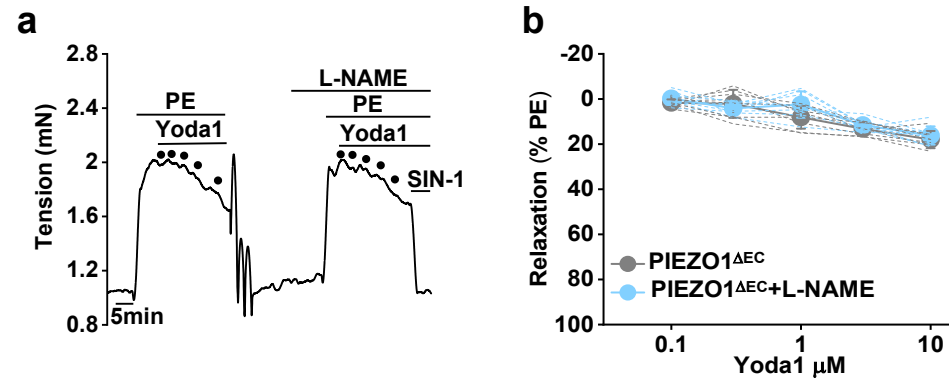

SI Figure S3

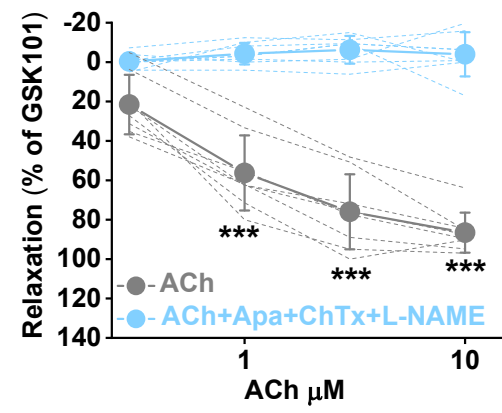

# SI Figure S4

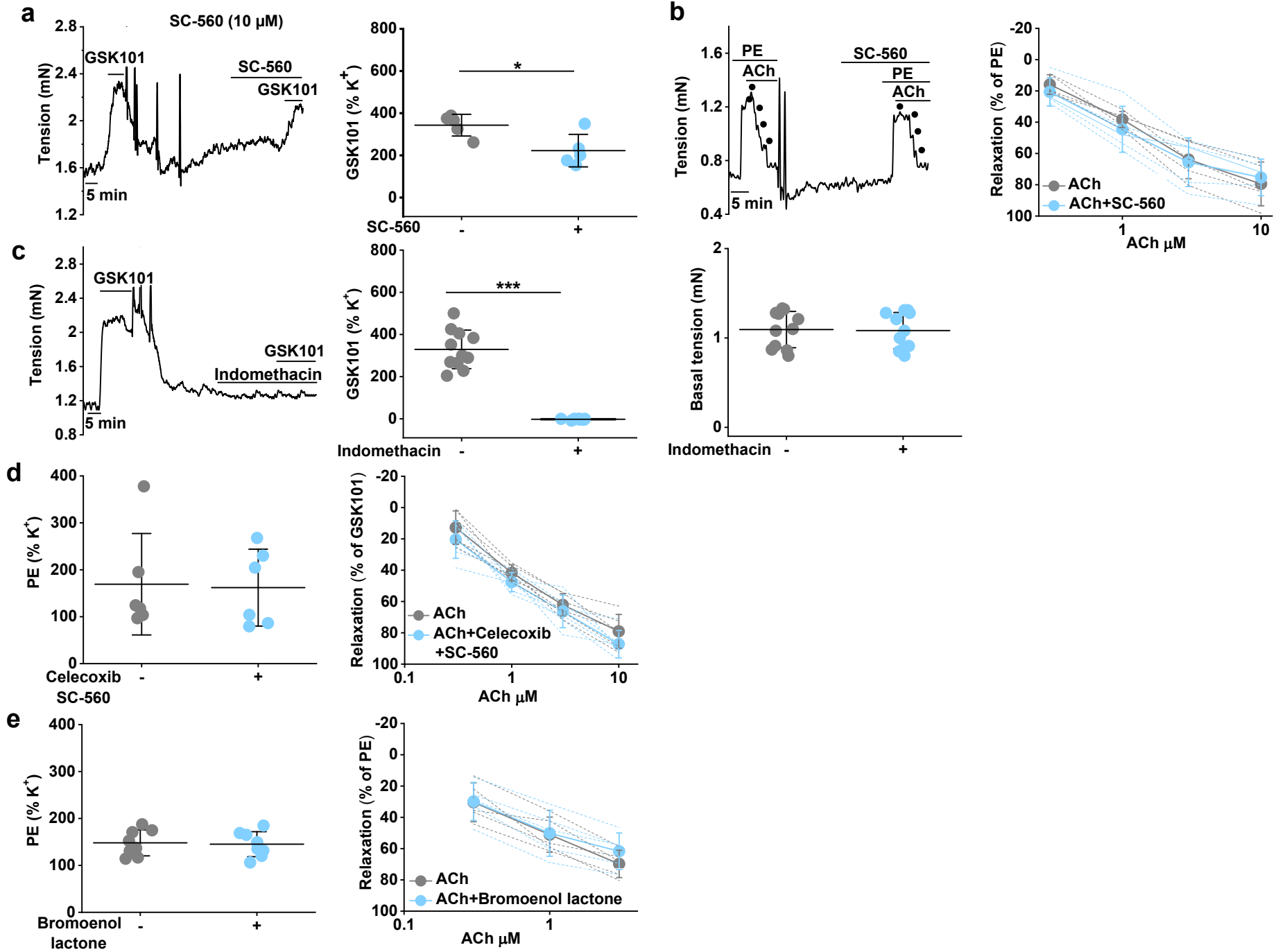

SI Figure S5

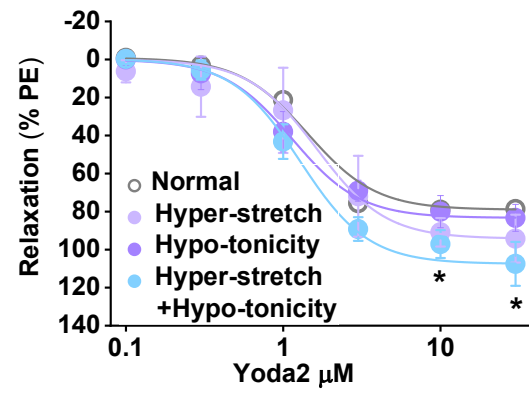

# SI Figure S6

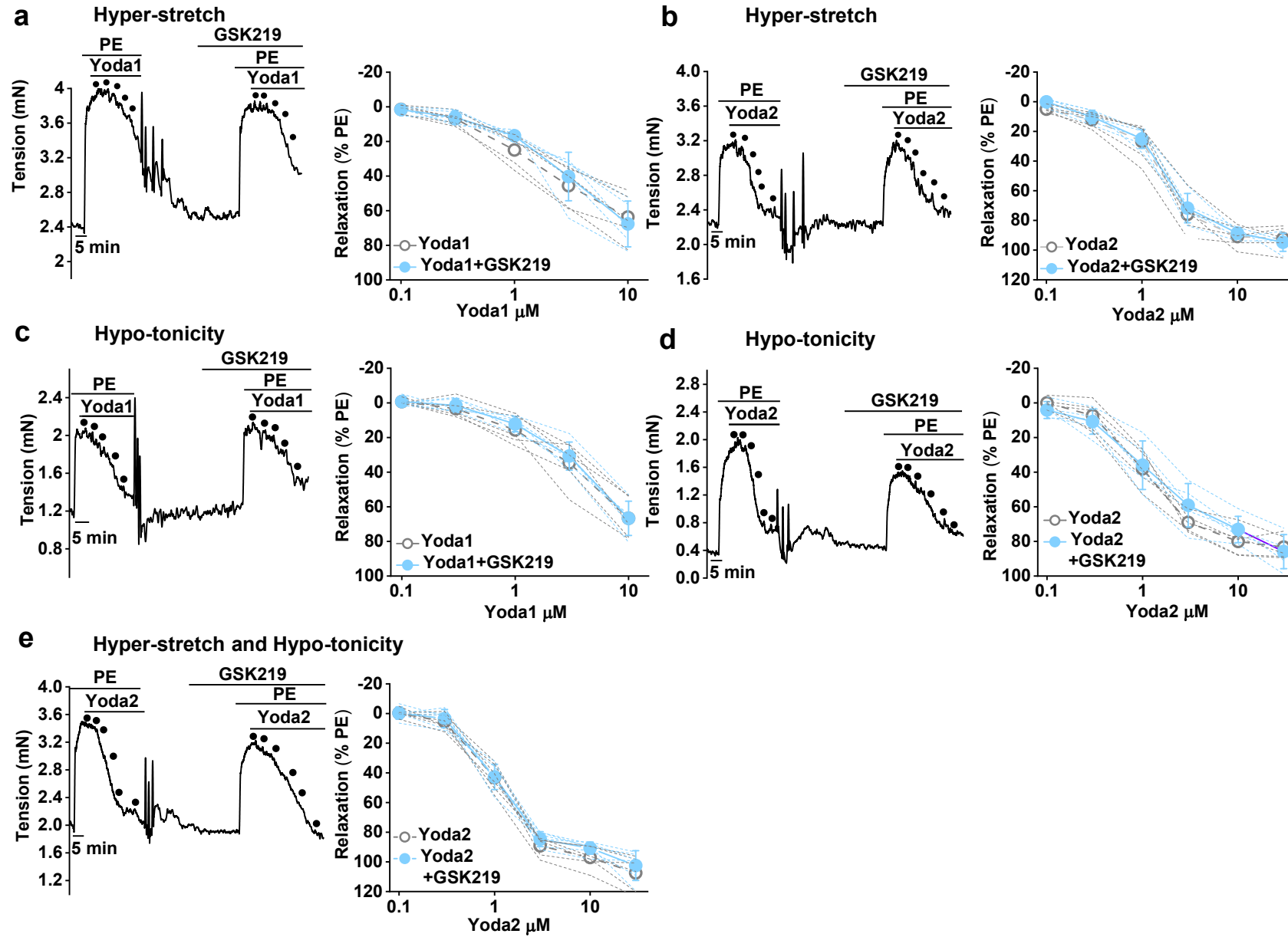

SI Figure S7

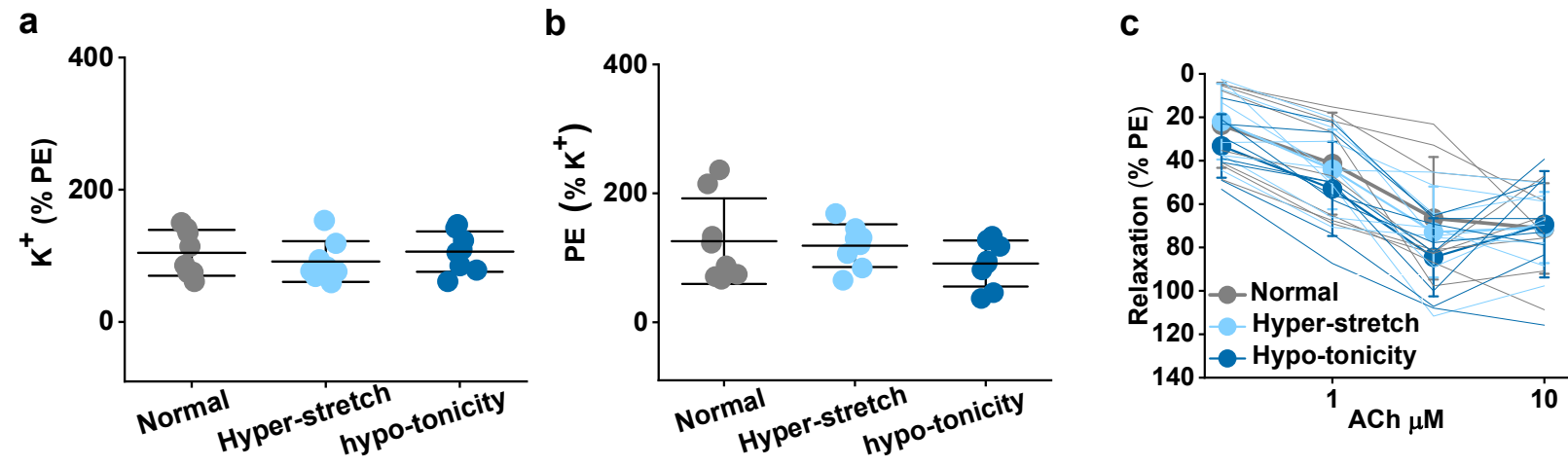

**SI Figure S8**

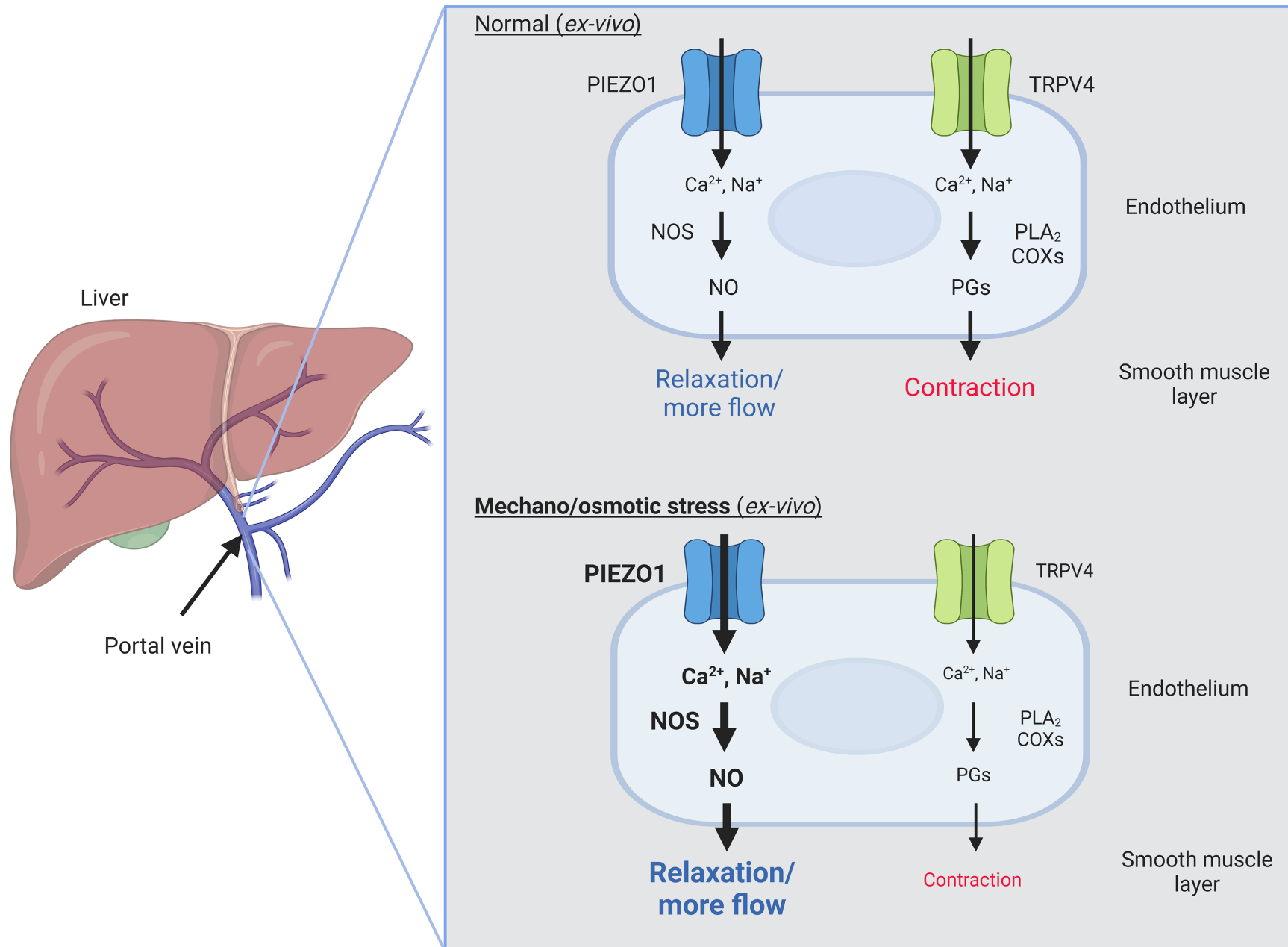

Supplement: Supplementary file 1 — Figure S1‐S8 [file LIV-43-2026-s001.pdf]
